# Upconversion Nanostructures Applied in Theranostic Systems

**DOI:** 10.3390/ijms23169003

**Published:** 2022-08-12

**Authors:** Chao Lu, Etienne Joulin, Howyn Tang, Hossein Pouri, Jin Zhang

**Affiliations:** 1 Department of Chemical and Biochemical Engineering, University of Western Ontario, London, ON N6A 5B9, Canada; 2 School of Biomedical Engineering, University of Western Ontario, London, ON N6A 5B9, Canada

**Keywords:** upconversion nanomaterials, theranostic system, lanthanide-doped upconversion nanoparticles, triplet-triplet annihilation upconversion, biosensing, drug carriers, NIR-triggered drug delivery, metal-organic frameworks

## Abstract

Upconversion (UC) nanostructures, which can upconvert near-infrared (NIR) light with low energy to visible or UV light with higher energy, are investigated for theranostic applications. The surface of lanthanide (Ln)-doped UC nanostructures can be modified with different functional groups and bioconjugated with biomolecules for therapeutic systems. On the other hand, organic molecular-based UC nanostructures, by using the triplet-triplet annihilation (TTA) UC mechanism, have high UC quantum yields and do not require high excitation power. In this review, the major UC mechanisms in different nanostructures have been introduced, including the Ln-doped UC mechanism and the TTA UC mechanism. The design and fabrication of Ln-doped UC nanostructures and TTA UC-based UC nanostructures for theranostic applications have been reviewed and discussed. In addition, the current progress in the application of UC nanostructures for diagnosis and therapy has been summarized, including tumor-targeted bioimaging and chemotherapy, image-guided diagnosis and phototherapy, NIR-triggered controlled drug releasing and bioimaging. We also provide insight into the development of emerging UC nanostructures in the field of theranostics.

## 1. Introduction

Theranostic systems refer to the systems that combine therapeutics and diagnostics and have the ability to monitor the efficacy of drugs after treatment, simultaneous treatment and diagnosis for patients and the ability to personalize and target treatments for patients [1]. The treatment of these diseases involves the delivery and release of the therapeutic drugs to the targeted sites. These delivered reagents can be chemotherapeutics (doxorubicin and docetaxel), proteins, genes and photodynamic and photothermal compounds [2,3,4,5]. The diagnostics part of theranostic systems involves the imaging-guided diagnosis to show the presence and track the status of diseased areas. These systems have shown potential in the treatment and diagnosis of diseases such as cancer, myocardial infarction and local tuberculosis [6,7,8,9].

The most commonly used bioimaging reagents in theranostic systems are semiconductor quantum dots (QDs). These semiconductor nanocrystals can exhibit Stoke-shifted (down-conversion) photoluminescence [2], which produces traceable signals that can be analyzed and used in various bioimaging and biolabeling applications [10]. However, QDs and other down-conversion nanoparticles (NPs) are limited by the fact that they require ultraviolet (UV) or visible excitation light to produce photoluminescence. The limitations and risks of using UV or shorter-wavelength radiation include: (1) shallow tissue-penetration [11,12]; (2) poor signal-to-noise ratio (SNR) because of light scattering and autofluorescence in the regions being penetrated (surface level contains skin and hair); and (3) damage or even death of the tissue and surrounding cells caused by the prolonged exposure to short-wavelength radiation [2,5].

**Figure 1:**
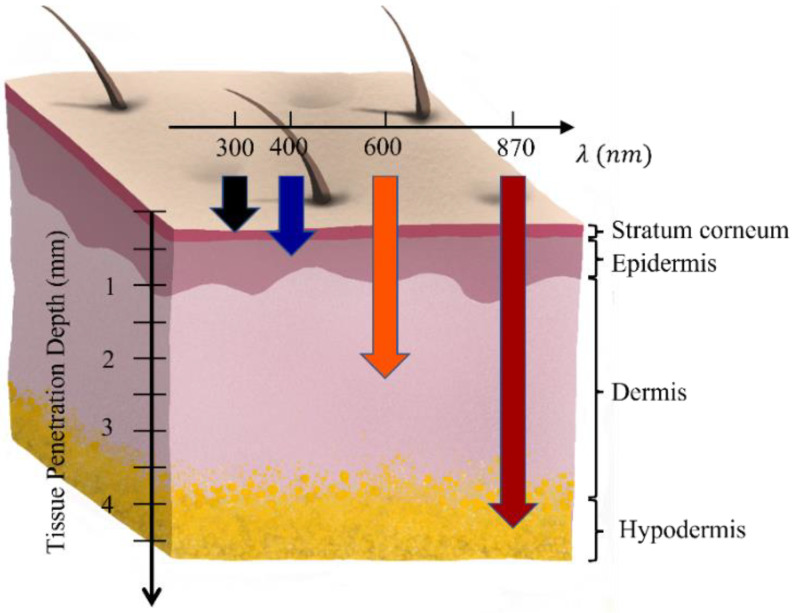
Skin penetration depth of light with different wavelengths [11,12].

**Figure 2:**
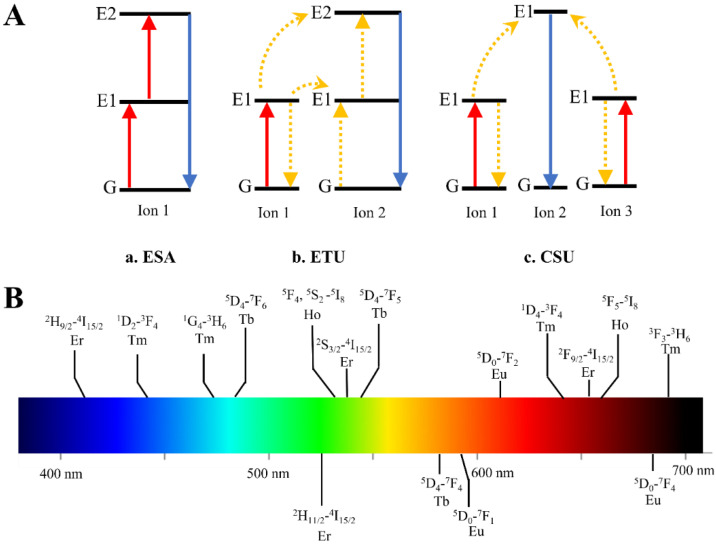
(**A**) Scheme of upconversion mechanisms. (**a**). ESA: Excited State Absorption, (**b**). Energy Transfer upconversion, (**c**). Cross Relaxation. G: ground state. E1: intermediate state. E2: excited state. Red line: photo excitation process. Blue line: emission process. Yellow dashed line: energy transfer process. (**B**) The wavelength of the emitted photons from the transition of lanthanide ions can be varied from 400 nm to 700 nm (as is shown in color bar), which shows the tunability of different lanthanide ions (reproduced with permission from [16]).

Recently, new nanostructures with upconversion luminescence (UCL) properties have been developed, which could convert low-energy excitation light, e.g., near-infrared (NIR), to high-energy emissions [2]. The applications of upconversion (UC) nanostructures have many advantages over other nanostructures, e.g., (1) deep tissue-penetrated NIR excitation being in the range of the “optical transparency window” of biological tissues (700–1100 nm), as shown in Figure 1; (2) good SNR due to reduced light-scattering and the absence of autofluorescence in the NIR excitation range, which significantly improves detection sensitivity; and (3) reduced photodamage to the cells and tissues of NIR [2,3,5]. Lanthanide (Ln)-doped UC nanostructures with a crystal matrix are good candidates for imaging and drug/gene delivery applications due to properties such as non-blinking, good biocompatibility, low cytotoxicity and minimized photobleaching [2,3,5]. The ease of surface modification and functionalization allows for the conjugation to a variety of specific biomolecules or reagents for chemotherapy, gene therapy, photodynamic therapy (PDT), photothermal therapy (PTT), protein-based therapy and sonodynamic therapy [2,13,14]. Organic molecular-based UC nanostructures, by using the triplet-triplet annihilation (TTA) UC mechanism, show a high UC quantum yield, a low requirement on the excitation power and one peak light emission, and they are also suitable for theranostic applications.

In this review paper, we present the UC mechanisms in Ln-doped UC nanostructures and the TTA UC mechanism with organic sensitizer and annihilator molecules in nanostructures. The hydrophobicity of UC nanostructures is the major barrier in applying them in aqueous environments for theranostics. The design and fabrication of hydrophilic and biocompatible Ln-doped UC nanostructures and TTA UC-based UC nanostructures have been reviewed and discussed. In addition, the current progress in the application of these well-designed UC nanostructures for diagnosis and therapy has been summarized, e.g., tumor-targeted bioimaging and chemotherapy, image-guided diagnosis and phototherapy, NIR-triggered controlled releasing and bioimaging. Finally, new nanostructures, which show special UC properties, are discussed. Insight into the emerging TTA-based UC nanostructure for theranostic applications has been provided.

## 2. Current Progress in the Design, Synthesis and Surface Modifications of UC Nanostructures

### 2.1. Lanthanide (Ln)-Doped UC Nanostructures

#### 2.1.1. Structures, Properties and Upconversion (UC) Mechanisms of Ln-Doped UC Nanostructures

Ln-doped UC nanostructures can emit higher energy photons by the absorption of two or more excitation photons with lower energy. UC processes in Ln-doped UC nanostructures are possible due to the unique electron configuration of Ln^3+^ (4*f*^n^ 5d^0−1^). The shielding of 4*f* electrons by outer and complete 5s and 5p shells permits 4*f*-4*f* transitions that establish long-lived energy states [15]. The long-lived nature and ladder-like structure of energy levels in Ln ions favor sequential photon absorptions and energy transfers between energy states, which are necessary for UC processes to take place [2]. Three common upconversion mechanisms have been introduced, i.e., the excited state absorption (ESA), energy transfer upconversion (ETU) and cooperative sensitization upconversion (CSU) mechanisms (as shown in Figure 2).

In the ESA process, an ion is excited from the ground state G to its first energy level E1 by absorbing one pump photon; then, it is promoted to its second energy level E2 by the sequential absorption of another pump photon. At this higher energy state, the ion can jump back down to the ground level by emitting a higher-energy (upconverted) photon. Unlike ESA, which comprises a single Ln^3+^ ion, two ions are involved in the ETU process [4]. First, a pump photon excites ion 1 (sensitizer) from the ground state G to its first energy level E1. The energy is then transferred to ion 2 (activator), bringing ion 2 to its first energy level E1 and causing the sensitizer to relax back to the ground state G. This process occurs a second time to bring Ion 2 to its second energy level E2. The activator finally relaxes and emits a higher-energy photon. The ion pairs of Yb^3+^/Tm^3+^, Yb^3+^/Er^3+^ and Yb^3+^/Ho^3+^ follow such mechanism when excited by a 975 nm laser [2,17,18]. CSU involves three ion centers, in which ions 1 and 3 are promoted to their first energy level E1 by absorbing a pump photon. They then simultaneously transfer their stored energy to ion 2, promoting it to its second energy level E2. A higher-energy photon is emitted when the excited ion 2 returns to its ground state G. The CSU process occurs more often in the Ln ion (such as Tb^3+^ and Eu^3+^) without metastable levels as an energy storage reservoir [18].

An improved UCL signal is always desired for the bioimaging diagnosis for theranostics. However, the surface quenching effect is the main cause of an impaired quantum yield and reduced emissions efficiency. The energy of the sensitizer on the surface is relaxed non-radiatively and transferred to surface defects, impurities, ligands and solvents [19]. Based on this, surface passivation would be useful in boosting the emission efficiency by forming a core-shell structure. This shell material can either be a core-similar inorganic matrix, such as NaYF_4_ and NaGdF_4_ [19,20], or another passive material, such as CaF_2_ and SiO_2_, which increase efficiency by 690-fold and stabilize UCNPs in an aqueous solution for 40 days, respectively [21,22].

**Figure 3:**
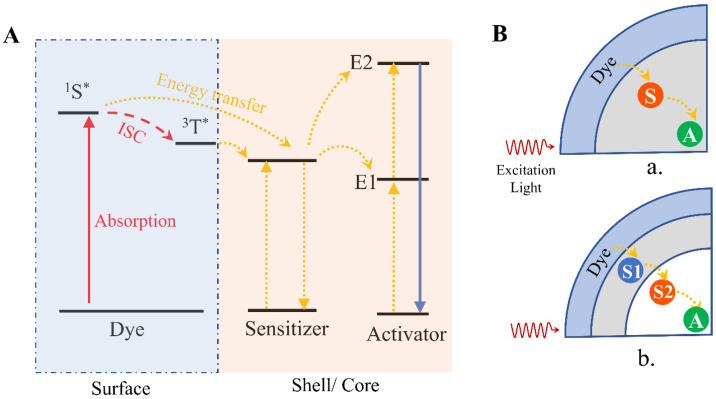
Dye-sensitized upconversion mechanisms and the typical structure of dye-sensitized upconversion (UC) nanostructures. (**A**) Schematic illustrations of dye-sensitized upconversion (^1^S*: single state, ^3^T*: triplet excited state, yellow line: nonradiative energy transfer, blue line: upconversion emission). (**B**) (**a**) Typical structure of dye-sensitized UC nanostructures in which only one type of sensitizer is contained. (**b**) Typical structure of dye-sensitized core/shell UC nanostructures, in which two different sensitizers were used (S: sensitizer, A: activator) (reproduced with permission from [23]).

On the other hand, in typical Ln-doped UC nanostructures, the doped Yb^3+^ ions work as sensitizers to absorb infrared radiation; then, the energy is transferred to activators (Er, Ho, Tm) by a nonradiative process. UCL is generated when the excited activator returns to its ground state. However, Yb ^3+^ ions, as sensitizers, could only absorb light in a narrow range from 938 nm to 974 nm [23]. The parity-forbidden nature of 4*f* electronic transitions in Ln ions leads to a relatively low extinction coefficient [24,25]. These limitations could be addressed when considering using organic dyes as sensitizers. A typical organic dye has a relatively broad spectral window (10 times broader) and a large absorption cross-section (around 1000 to 10,000 times higher than Yb^3+^) [23,25]. Optical properties can also be tuned by the synthetic techniques, and organic dyes can easily be engineered on the surface of UC nanostructures by covalent bonding, electrostatic attraction or physical absorption. The excitation wavelength of NaYF_4_: Yb, Er UC nanostructures was successfully shifted from the optogenetic neuron excitation window to a biocompatible and deep tissue-penetrable 800 nm wavelength through the sensitization of dye IR 806 [26]. The UCL of NaYF_4_:20%Yb,2%Er@NaYF_4_:20%Nd can be largely enhanced by 600-fold by dye IR 808 sensitization [27]. The organic dyes on the surface of UC nanostructures are able to broadly and strongly harvest NIR light and excite to the single state (^1^S*) and the triplet excited state (^3^T*) by ISC. Then, the energy is transferred to Ln^3+^ on the surface of Ln-doped nanoparticles by nonradiative energy transfer processes. The upconversion process in the inorganic core would follow ETU mechanisms (Figure 3A). These processes can happen on either UC core nanostructures or core/shell structures (Figure 3B).

#### 2.1.2. Synthesis Processing of Ln-Doped UC Nanostructures

Various methods have been developed for the preparation of Ln-doped UC nanostructures, such as sol-gel, pyrolysis, thermolysis, coprecipitation and hydrothermal/solvothermal methods. Ostwald-ripening, cation exchange reaction, seed-mediated heat up and successive layer-by-layer methods are methods to prepare Ln-doped UC nanostructures with core-shell nanostructures [2,4,28,29]. The Ostwald-ripening and seed-mediated heat up methods both need a preformed UC nanostructure core as a seed nucleus, followed by the deposition of shell precursor material on the surface. Shell thickness can be controlled in the Ostwald ripening method by adjusting stoichiometric quantities of the sacrificial nanoparticles or by the continuous injection of shell monomers [2,29]. In comparison, the seed-mediated heat up method typically forms non-uniform shells [30]. The cation exchange reaction method is a straightforward way to produce a core-shell structure and is carried out by exchanging cations on the UC nanostructure’s surface with surrounding free cations in a reaction solution, leading to a heterogeneous shell layer [28,29]. The successive layer-by-layer method is an alternative method to the seed-mediated method, whereby shell thickness can be easily controlled [29,30].

Sol-gel is the typical method used to synthesize UC oxide powders, such as Y_2_O_3_ [31]. In the pyrolysis and thermolysis methods, metallic precursors are first dissolved in a high-boiling-point solvent, followed by thermal decomposition. Lastly, they undergo crystallization and the formation of UC nanostructures [2,3,5,31]. These two methods apply high pressures and temperatures in a sealed vessel to produce pure and highly crystalline products. Additionally, capping ligands such as oleic acid, trioctylphosphine oxide (TOPO) and oleic acid (OA) are added to prevent agglomeration. Coprecipitation involves the precipitation reaction of positively and negatively charged ions in a homogenous solution to form uniform UC nanostructures and is one of the most simple and convenient methods for the synthesis of Ln-doped UC nanostructures.

#### 2.1.3. Surface Modification of Ln-Doped UC Nanostructures for Theranostic Applications

Surface modifications are necessary to improve the hydrophilicity and biocompatibility of UC nanostructures and functionalize their surface for practical use in theranostic systems. UC nanostructures are typically insoluble in water due to the hydrophobic organic capping ligands (i.e., OA and TOPO) on their surface. This significantly limits their applications in aqueous conditions, where water dispersibility is imperative [2]. Furthermore, it is difficult to directly conjugate UC nanostructures with target biomolecules or bio-receptors due to the limited functional groups on the UC nanostructures’ surface. This hinders their application in bioimaging for diagnosis, targeted delivery for therapeutics and specific molecular detection [2,5]. A combination of hydrophobic and hydrophilic ligands can be used to cap UC nanostructures in the coprecipitation method and hydrothermal/solvothermal techniques, which can, to an extent, reduce the need for surface modifications to improve water dispersion. Altogether, the selection of ideal synthesis techniques and surface modifications of UC nanostructures can help to optimize their biocompatibility and water solubility and reduce their cytotoxicity for theranostic applications. Several strategies for the modification of UC nanostructures are described below.

Silica-coated UC nanostructures can be used to form core-shell structures, the surfaces of which are easily modified by bio-receptors for targeted drug delivery. This silica shell can not only increase the hydrophilic and biocompatible properties of UC nanostructures but also has no influence on the photoluminescence of UC nanostructures [32]. A modified Stöber method is used to form a layer of silicon dioxide of controlled thickness. UC nanostructures are dispersed in an ethanol and water solution, and a silane precursor (i.e., tetraethyl orthosilicate) is hydrolyzed and polymerized on the nanoparticle surface [31,32].

Ligand exchange is a widely investigated method for surface modification. The newly introduced ligands should have a stronger coordination ability with respect to the cores than the existing ligands [31,32]. Citric acid [33], poly(acrylic acid) [34,35], polyethyleneimine (PEI) [36,37,38,39], hyaluronic acid [14], 3-mercaptopropionic acid [40] and 11-mercaptoundecanoic acid [41] were reported to successfully stabilize UC nanostructures and increase water solubility. Similarly, it has also been widely applied for surface modification by the utilization of the ligand–ligand interactions. The hydrophobic–hydrophobic interaction involves the van der Waals interaction between the hydrophobic segment of amphiphilic molecules and the capped hydrophobic ligand. These amphiphilic molecules can be surfactants (TWEEN [42]), synthetic polymers (PEG [17]), natural polymers (polysaccharide polymers [39,43]) and proteins (transferrin [44]). The outer hydrophilic segment increases the water solubility and provides active sites for bioconjugation, while the hydrophobic area on the surface provides loading sites for hydrophobic drugs such as doxorubicin (DOX). In addition, ligand-free UC nanostructures with good water dispersity can be obtained by treating OA-capped UC nanostructures with strong acid or sonication under ethanol. The strong coordination ability of ligand-free nanoparticles allows them to be modified by various organic functional groups, including –COOH, –NH_2_ and –OH.

### 2.2. Organic Molecular-Based UC Nanostructures Using the Triplet-Triplet Annihilation Upconversion Mechanism

#### 2.2.1. Triplet-Triplet Annihilation-Based Upconversion Nanostructures

Organic triplet-triplet annihilation (TTA)-based upconversion (UC) nanostructures are reported to be excellent carriers in the fields of bioimaging and photoactivated drug release due to their good biocompatibility, low excitation power density, high upconversion quantum yield and tunable excitation/emission wavelength [45,46,47,48,49]. TTA-based UC nanostructures are capable of being excited at a low excitation power [50]. A TTA UC sensitizer and annihilator pair was reported by Radiy et al., which could be excited by a commercial green laser at low power (<5 mW, 532 nm), and the resulting luminescence emission would be bright enough to be observed with the naked eye [51]. Excitation and emission wavelengths can be easily tubed by the selections of the triplet sensitizers and acceptors [52].

**Figure 4:**
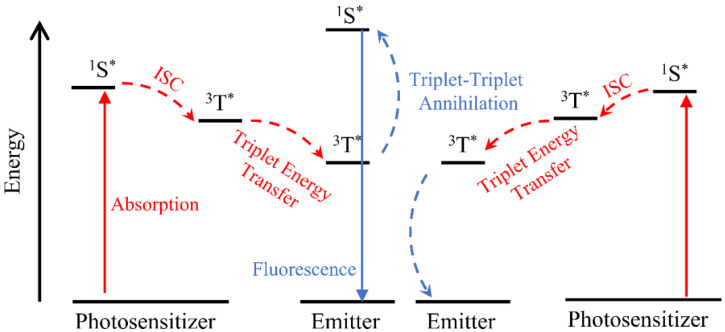
Scheme of the anti-stokes process of triplet-triplet annihilation upconversion nanoparticles (^1^S*: single state, ^3^T*: triplet-excited state, ISC: intersystem crossing process).

In this anti-stokes process, the photosensitizer is first excited from the ground state to the single state (^1^S*) by the absorption of the photo with low energy. The triplet-excited state (^3^T*) is then achieved by the intersystem crossing process (ISC). Because of the long lifetime of triplet-excited state, the triplet energy of the sensitizer is transferred to the annihilator by the triplet energy transfer process [53]. When the two sensitized annihilator triplets collide, a higher energy singlet excited state will be made and generate upconversion fluorescence (Figure 4) [54,55]. TTA-based UC nanostructures, which could absorb NIR light, have their advantages in biological applications, such as deep tissue penetration and low photo-induced damage for the organism [56,57]. The pair of Pd(II) phthalocyanine and rubrene is an example of a photosensitizer and emitter and was reported to be excited by a 725 nm laser and generate 635 nm light [58].

#### 2.2.2. Design of TTA-UC-Based Nanostructures for Theranostic Applications

Most of the sensitizer and annihilator pairs are hydrophobic, and those TTA UC processes occur in the organic phase [59], which limits their application in biological environments. It is important to manipulate other nanostructures, such as polymeric NPs, dendrimers and silica NPs, to load TTA pairs in the aqueous solution and keep their UC properties.

Biocompatible silica nanoparticles are a suitable matrix for loading TTA pairs in their hydrophobic core, and the shell can be modified by an amphiphilic polymer, such as Pluronic F127 [60]. It showed a good luminescence quantum yield (as high as 4.5%) in an aqueous solution and could be used as a labeled fluorescent probe for the in vivo imaging of a living mouse. Micelles could also be directly formed by the self-assembly of an amphiphilic polymer, which is a straightforward way to lade the sensitizer and annihilator pair. For example, poly (propylene oxide) segments of Pluronic P123 could form the hydrophobic core to load the octaethylporphyrin Pd complex (sensitizer, PdOEP) and 9,10-diphenylanthracene (annihilator, DPA), while poly (ethylene oxide) segments form the hydrophilic shell [40]. Similarly, an oil-in-water-in-oil-in-water (O1/W1/O2/W2) triple-emulsion method can also be applied, in which organic molecule-based TTA pairs are dissolved in the innermost core oil phase (O1), and the polymeric shell phase (O2) provides structural support. A microfluid device was reported by Jae-Hyuk et al. to produce emulsion microcapsules, and the capsules showed good uniformity with a 160 μm diameter [61]. In addition, TTA pairs and various monomers, such as styrene, methyl methacrylate and acrylic acid, were dissolved in the organic phase together, followed by vigorous stirring and sonication to form the microemulsion system. Polymeric nanoparticles could be formed when these monomers were polymerized [62,63].

## 3. Current Process for UC Nanostructures for Theranostic Applications

### 3.1. Multifunctional Carriers for Therapy, Diagnosis and Bioimaging

UC nanostructures have been well-studied and characterized in terms of their ability to act as drug carriers and UCL imaging agents. UC nanostructures serve multifunctional purposes: (1) Upconversion emission light with a large anti-Stokes shift showed a better SNR for bioimaging; (2) Their surface can be modified to carry therapeutic reagents for chemotherapy, gene therapy, immune therapy, sonodynamic therapy, photo thermal therapy (PTT) and photo dynamic therapy (PDT); (3) Ligands and aptamers can be conjugated on the surface of nanostructures to target specific cells or sites of the body. In this section, their theranostic applications will be discussed.

As mentioned in Section 2, Ln-doped UC nanostructures are typically capped with OA, TOPO and other hydrophobic ligands after synthesis, which impair their hydrophilicity and increase the cytotoxicity. Ligand exchange and hydrophobic–hydrophobic interaction between ligands are effective strategies to replace or modify the hydrophobic ligands with amphiphilic or hydrophilic ligands in order to increase the biocompatibility and water solubility of UC nanostructures for biomedical applications. Various polymers, such as poly(acrylic acid) (PAA) [64], terminal functionalized PEG [65,66,67], di-block copolymer poly(ethylene oxide) (PEO)-polymethacrylate (PMA) [68] and polyvinylpyrrolidone (PVP) [66], could replace the original ligands to form a denser polymer layer. The amphiphilic molecules, such as TWEEN [42], chitosan [39], guar gum [43] and transferrin [44], could interact with the ligands (e.g., PEI, OA) via van der Waals force to form a hydrophilic outer layer. Both of these strategies not only increase the dispersibility in an aqueous solution but also provide many sites for drug loading by electrostatic interactions (nucleic acid-based drugs and positive charged polymers [69]), direct physical conjugation (hydrophobic interactions between lipophilic molecules and the hydrophobic polymeric segment [67]) and the covalent bonding of drugs to polymers [70].

However, the UCL of NaYF_4_:Yb,Er and NaYF_4_:Yb,Tm was reported to be quenched by the use of PAA as a shell layer [71], and oleate-capped UC nanostructures were found to lose their ligands when carboxylate groups were protonated in the acid environment [72]. Hence, the direct encapsulation of UC nanostructures into micelles by the self-assembly of amphiphilic polymers could be an alternative for the ligand exchange method. The unmodified UC nanostructures could be loaded with lipophilic dyes or drugs in the hydrophobic core while the hydrophilic segments of amphiphilic polymers form the shell to provide water solubility [73]. It is also feasible to incorporate UC nanostructures with other matrices to work as carriers. A silica shell can be formed around the core of Ln-doped UC nanostructures with hydrophobic ligands. The mesoporous shell structure has been demonstrated as feasible for drug loading [13,74,75,76,77]. In addition, metal-organic frameworks (MOFs) are crystal materials with a two- or three-dimensional periodic structure which is formed by the coordination-driven assembly of metal ions or clusters and organic ligands [78,79]. A large specific surface area, a highly ordered porous structure, structural flexibility and good biocompatibility enable MOFs to achieve a high loading efficiency and to be ideal nanoplatforms for biomedical applications [80,81,82,83]. The coordination or electrostatic interactions between UC nanostructures and metal ions allow for the in situ formation of MOFs around the UC nanostructures [84,85,86]. Other carriers such as nanodiamonds (NDs), a carbon-based material, can incorporate with UC nanostructures to work as a carrier [87]. These strategies all make UC nanostructures-based carriers feasible for loading drugs and dyes for the therapeutics applications.

Therefore, these UC nanostructures have good potential to work as carriers for the therapy of various diseases. DOX is a generally used chemotherapy reagent to kill tumor cells and has been reported to be encapsulated into the hydrophobic zone of polymer-coated surfaces [42,88], the hydrophobic core of liposomes [89] and MOFs [90]. Similarly, the platinum(IV) prodrug can also be loaded in these sites for chemotherapy [13,36,37,38], siRNA can be conjugated with the surface of PEG-modified UC nanostructures for gene therapy [91] and docetaxel can be loaded into the liposomes with UC nanostructures to treat glioma in the brain [92]. In addition, the mesoporous silica shell and MOFs have been reported as good carriers for PDT reagents (e.g., Rose Bengal, zinc(II)phthalocyanine and chlorin e6) [75,76,77,86], which will be discussed in the next section.

On the other hand, the diagnosis of diseases such as cancers is an important issue for worldwide public health. UCL imagining is a promising method in targeted sensing and cell imagining because of the excellent sensitivity and spatial resolution [31,80,93,94]. If UC nanostructures-based carriers could recognize and attach to the specific targets, followed by being triggered by NIR light, UCL would be achieved to visualize targeted sites, thus realizing the diagnosis of various diseases. For example, cancer cells can be distinguished from normal cells based on the difference between their cell membranes. Certain proteins on the cell membrane can be used as biomarkers, such as overexpressed human transferrin receptors and overexpressed folate receptors [95,96]. After conjugating the related ligands or aptamers on the well-designed UC nanostructure-based carriers, they could not only delivery chemo- or photo-therapy agents to the targeted sites but also visualize these sites for disease diagnosis. The theranostic applications of UC nanostructures-based carriers are summarized in Table 1. Transferrin, an antibody-binding protein (Protein G) folic acid can be conjugated to these carriers to make them more specific to tumor cells and give the direct visualization of the tumor for diagnosis [77,92,97]. In addition, the more acidic nature of tumor microenvironments contributes to lower pH environments compared to the normal cell, which promotes the electrostatic interaction between positively charged chitosan-coated UC nanocarriers and negatively charged tumor cell membranes and achieves tumor-specific accumulation [39]. At the same time, the loaded chemo-therapy agents, e.g.*,* DOX, provide a therapeutic effect. Recently, Ling et al. have designed a pH-controlled dual drug delivery system that combines MOFs and UC nanostructures. At low pH values (6.0 and 5.0), the MOFs collapse, releasing DOX and 5-fluorouracil into the tumor environment, which proved to have a synergistic effect and greater cytotoxicity. In essence, it was also proved to be as useful as gated multi-drug carriers, while carrying bioimaging potential as *T*_1_ MRI contrasting agents [98].

### 3.2. NIR-Driven Photodynamic and Photothermal Therapy and Diagnosis for Tumor Theranostics

The treatment of cancers is not limited to the transportation of chemotherapy agents, which have nonspecific toxicity and easily get accumulated to various organs [113]. PDT is a promising method for cancer treatment because of the low side effects and high selectivity [93]. The interaction between UV or visible light and photosensitizers (PS) could generate cytotoxic reactive oxygen species (ROS), which have been proved to oxidize amino acid residues, inhibit tumor cells and restrict the self-assembly of neurotoxicity proteins [114,115,116]. However, unlike NIR light with high tissue penetration ability, UV and visible light have shallow tissue penetration depth, which confines the PDT for shallow-seated tumors [117]. In order to make the PDT feasible for deep-tissue application, UC nanostructures, which could convert low-energy NIR light to high-energy visible or UV light, are introduced to incorporate with the photosensitizers. This well-designed system may become a good solution for tumor theranostics because PDT agents have a good therapeutic effect to inhibit tumor cells, and UC nanostructures could convert NIR light to visible or UV light to trigger photosensitizers for PDT and have a potential function as bioimaging agents for tracking and visualizing tumors for disease diagnosis.

**Figure 5:**
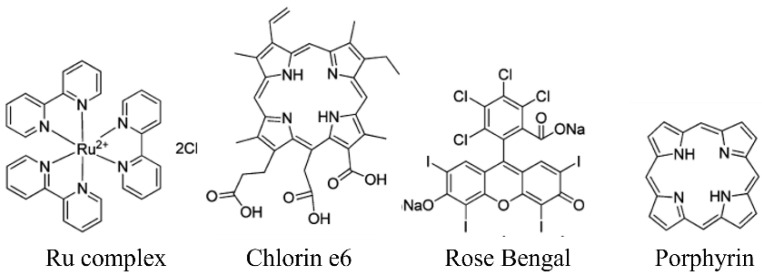
Chemical structures of photosensitizers for photodynamic therapy (PDT) (add MB and ZnPc).

**Figure 6:**
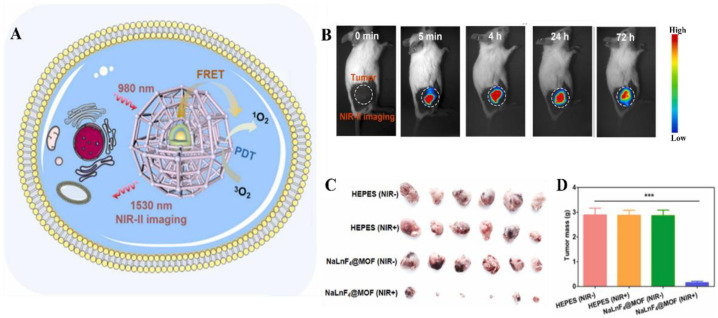
(**A**) Schematic illustration of NaLnF_4_@MOF in a cell for NIR-triggered photodynamic therapy and NIR-II bioimaging for diagnosis. (**B**) NIR-II images of BALB/c tumor-bearing mice treated with NaLnF4@MOF nanoparticles after intratumoral injection (0 min, 5 min, 4 h, 24 h and 72 h). (**C**) The images and (**D**) mass of excised tumors after two weeks post-injection (HEPES is the buffer solution as a control, *** *p* < 0.001) (Reprinted with permission from [84]).

In the NIR-driven PDT theranostic system, the UC nanostructures are triggered by NIR light and convert light with low energy to light with high energy. Then, this high-energy light interacts with PS to generate ROS, e.g., ^1^O_2_ and OH, to kill cancer cells. Chlorine6 (Ce6), rose engal (RB), porphyrin, MnO_2_ and Ru complexes can be used as photosensitizers for PDT (the chemical structures are shown in Figure 5) [44,84,86,99,118]. There are various conjugations of these hydrophobic photosensitizers, such as Ce6 and RB, with UC nanostructures: (1) Encapsulation inside the hydrophobic core of phospholipids [112]; (2) Conjugation inside the frame of MOFs [86]; (3) Encapsulation between the UC core and silica shell [75,76]; (4) The formation of cerium oxide and manganese dioxide [101,119]. These well-designed structures not only guarantee the utilization efficiency of upconversion emission light by photosensitizers but also keep their UCL properties for bioimaging, which were proven in in vitro imaging for HeLa cells, KB cells and REF52 cells and in in vivo imaging for mice. For example, a well-designed system showed an enhanced tumor inhibition rate of up to 95%, in which Er^3+^-doped NaLnF_4_ worked as a core material, converting 980 nm NIR light to yield green (525 nm and 542 nm) and red (655 nm) emissions for PS activation and 1530 nm emissions for NIR-II imaging. In situ-formed Zr-based porphyrin MOFs acted as a shell material and as PS to generate ROS [84]. After intratumoral injection, these UC nanostructured-based MOFs diffused rapidly and homogenously throughout the entire tumor, which provided visualized images to check the conditions of tumors for accurate therapy and diagnosis. After NIR treatment for 14 days of post-injection, the obvious tumor inhibition effect can be observed on the excised tumor sites (Figure 6). In addition, the immobilization of tumor-targeting antibodies on the hybrid nanocomposites composed of RB and UC nanostructures makes them more targeted to tumor cells, offering promise for image-guided diagnosis [77]. The targeted imaging and selective killing of human colorectal adenocarcinoma HT-29 cells by PDT were validated via in vitro experiments.

On the other hand, UC nanostructures can work as carriers for PTT, which utilize light-to-heat conversion through a nonradiative transition. Gold nanoparticles [120,121] and graphene and its derivatives [122,123] are good PTT reagents because of their strong visibility to near-infrared absorbance and biocompatibility. For instance, a gold shell can be grown on the surface of citric acid-modified NaYF_4_:20%Yb,2%Er@NaGdF_4_ NPs [33], and nanographene oxide sheets are reported to conjugate to PEGylated NaGdF_4_:20%Yb,2%Er@NaGdF_4_:25%Yb,25%Nd NPs via a covalent bond [106]. The tumor therapeutics of these two structures can be enhanced by the synergetic effect of DOX-loaded gold shell UC nanostructures (chemotherapy + PTT) [33] and cerium oxide- and nanographene oxide sheet-loaded systems (PTT + PDT) [106]. The UCL imaging was verified in Hela cells and U14 tumor-bearing mice. Interestingly, the resonant excitation ^2^F_5/2_ → ^2^F_7/2_ of Yb^3+^ was reported to be applied for PTT. After the tumor is labeled by NaYF_4_:18%Yb,0.6%Tm@NaYF_4_ NPs in vivo, the temperature gap between the tumor sites and the normal sites can reach 2 °C within 3 min of 975 nm radiation with an intensity of <1 W/cm^2^ [105]. The accumulation of these UC nanostructures inside the tumor was verified by the visualization of mice under 975 nm radiation.

### 3.3. NIR-Triggered Theranostic System

Most of the current drug delivery carriers are designed to manipulate ligands with a higher affinity to specific transporters, receptors or cell surfaces for targeted drug delivery [124,125]. Such strategy has been proven to be effective, but the immunogenicity of enzymes, the first pass effect from the liver and spleen and the untargeted cells with the same receptors for our ligands all lowered the delivery efficiency and caused many side effects. For example, albumin NPs conjugated with low-molecular-weight protamine are designed to bind to an albumin-binding protein (e.g., SPARC and gp60) on glioma and tumor vessel endothelium. However, the results from Lin et al. also showed that other reticuloendothelial systems, such as the lungs and the liver, are capable of capturing such albumin-labeled conjugations [126]. For this reason, it is important to introduce and design an on-demand system which is independent of the physiological environment and in which the release of biologically active molecules can be controlled by a remote or external stimulation, such as ultrasound, light, heat and a magnetic field.

**Figure 7:**
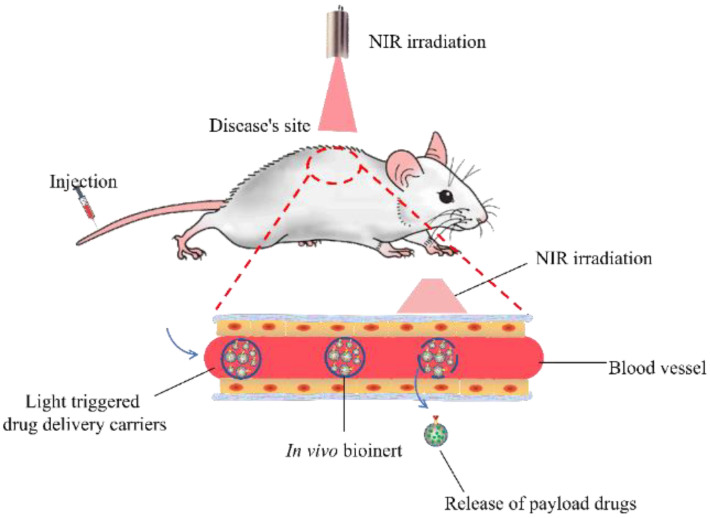
Scheme of the mechanism of the light-triggered drug vehicles (administered by intravenous injection and triggered by light when approaching the targeted sites).

Light has been extensively applied in various biomedical applications such as smart drug delivery, PDT [127] and the controlled degradation of tissue engineering scaffolds [128] because of its non-invasive nature, the ease of its use and control and its high spatial resolution. The design of light-responsive drug carriers is a good solution to boost delivery efficiency. The general structure of light-responsive carriers and the targeted transportation mechanisms are shown in Figure 7. This well-designed drug carrier has a core shell structure. The outer layer is composed of biocompatible polymers, the segments of which are linked by light immolative moieties. These layers are bioinert in vivo after administration and provide protection for the inside drug carriers in case of premature release and unnecessary consumption during blood circulation. They are stable and bio-inactive without light irradiation. Since they are composed of UV/visible light-responsive compounds, the chemical bond of them could be cleaved upon irradiation. The inside nanocarriers are ligand-modified carriers which may contain therapeutic reagents and UC cores or quantum dots for light-responsive bioimaging. An external light stimulus is applied on the specific site of the route. Once this designed carrier is passed by the tissue sites exposed to the light beam and triggered by light, the light-sensitive linkers in the outer layer will be broken down and destroy the compact layer structure, followed by the release of an affinity drug delivery system to target sites.

Molecules with ortho-nitrobenzyl moiety coumarin derivatives are readily cleaved upon UV irradiation and are suitable for such a delivery strategy [129,130,131,132,133], but the challenge of the in vivo utilization of radiation below 650 nm involves low tissue penetration, a requirement for expensive high-energy lasers and tissue damage. NIR, with its minimal absorption by tissue and deep tissue penetration (up to 10 cm through breast tissue and 4 cm of skull/brain tissue [134]), has been widely utilized as the excitation light source in bioimaging and light-stimulus drug carriers for in vivo applications. Considering the nature of the UCL of UC nanostructures, which involves converting photons with low energy to high energy by the anti-Stokes process, TTA-based UC materials and UC nanostructures can be employed to convert NIR light to UV or visible light and then trigger the photoreaction of light-sensitive materials [135].

**Figure 8:**
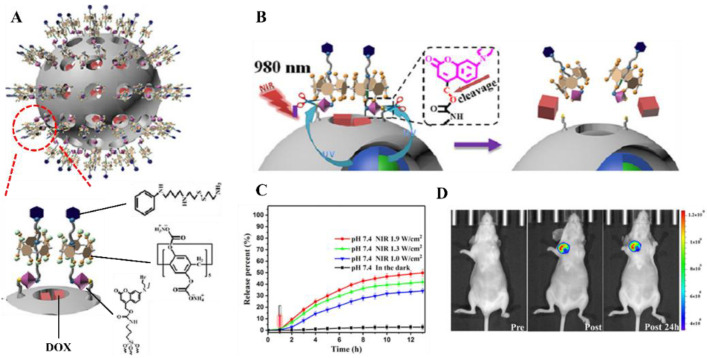
Schematic illustration of the NIR-triggered theranostic system and its effect on controlled release and image-guided diagnosis. (**A**) Structure of SiO_2_-coated upconversion (UC) nanostructures and the detailed structure of pore orifices anchored with supramolecular valves. (**B**) NIR-triggered release mechanism. (**C**) Doxorubicin (DOX) releasing profile under dark and different NIR irradiation dosages (1.0–1.9 W cm^–2^). (**D**) In vivo UCL imaging of a tumor-bearing Balb/c mouse: pre-injection, post-injection and 24 h post-injection (from the left to the right). Reproduced with permission from [137].

An NIR-triggered drug carrier was also designed based on the obvious structural change caused by the amphiphilicity change of copolymers. Cationic conjugated polyelectrolyte brush (CCPEB) not only encapsulated the UC nanostructure NaYF_4_:Yb/Tm but also was abundant in positive charges to carry the siRNA and photosensitizer [136]. Under the IR irradiation, the photodegradable 2-nitrobenzyl functional groups in the polymer structures were decomposed and formed the zwitterionic polymer, which promoted the release of siRNA up to 80%. This system was limited to therapeutic applications. Another well-designed NIR-triggered UC nanostructure-based carrier was fabricated for theranostics including drug releasing and tumor-targeted imaging [137]. The SiO_2_-coated NaYF_4_:40%Yb,0.5%Tm@NaGdF_4_:10%Yb nanocrystals were prepared first. The silica shell is hollow and mesoporous, with a pore size around 3.6 and 4.8 nm, which guarantees the high loading efficiency of the anticancer drugs, e.g., DOX. The NIR-responsive function was realized by anchoring supramolecular valves around the pore orifices (Figure 8A). The linker between the supramolecular valves and the pore surface was coumarin derivatives, which are light-responsive. These valves could efficiently block the release of DOX from the pores under the dark. Once irradiated by 980 nm NIR light, the UC nanostructure-based core could emit 345 nm and 360 nm light (UV region). The emission light with high energy would trigger the photocleavage of coumarin derivatives and open the supramolecular valves (Figure 8B). This releasing rate could be precisely controlled by the NIR irradiation power (Figure 8C). After being intratumorally injected into a tumor-bearing nude mouse, the NIR luminescent signal could even be detected from the tumor site after 24 h post-injection (Figure 8D), which made this system a candidate bio-probe for tumor diagnosis. In addition, Ru complexes can also work as photoactive molecular valves to control the drug release in mesoporous silica-coated UC nanostructure-based carriers [133].

### 3.4. Biocompatibility of UC Nanostructures

Most of the Ln-doped UC nanostructures are capped with modified ligands such as PEI, PAA, PEG, natural polymers and proteins or encapsulated into MOFs or liposomes to form a core shell structure with silica coating (summarized in Table 1). The safety of these UC nanostructures is largely dependent on the surface properties. The in vitro cytotoxicity of various Ln-doped UC nanostructures-based theranostic systems in Table 1 was evaluated, and all of them illustrated good cell viability and biocompatibility in the employed incubation conditions. For example, the nanostructures with silica coating were proven to not have overt toxicity via in vivo experiments in mice with a high dose for two weeks [138]. These in vitro cytotoxicity and in vivo acute toxicity studies may fail to directly reflect the potential chronic toxicity. Similarly, the cytotoxicity of silica-coated TTA-UC-based nanostructures did not show acute toxicity [139]. The safety concern for the organic molecular-based UC nanostructures using TTA UC mechanisms is that toxic co-solvents are essential to dissolve the TTA-UC dyes during the fabrication processes, but they are difficult to remove completely, which brings uncertainty in biological applications [140].

## 4. Future Perspective

NIR, with its minimal absorption by tissue and deep tissue penetration, has been widely utilized as the excitation light source in bioimaging, light-stimulus drug carriers and photodynamic therapy [139,141,142]. For this reason, Ln ions-doped inorganic UC nanostructures have also been widely investigated in photo-responsive drug release and fluorescence sensors in the past decades [143,144,145,146] because of their ability to convert photons from low energy to high energy to trigger the prodrug release. However, such UC nanostructures are highly dependent on a high excitation power density, and the relatively high toxicity remains a problem for their biomedical applications [147,148].

TTA UC-based sensors usually possess similar benefits to inorganic Ln-doped UC nanostructures such as minimal background interference, low photodamage and less light scattering [94,149]. Secondly, they have key advantages over Ln-doped UC nanostructures. Since TTA-based UC nanostructures rely on triplet-excited states of photosensitizers and annihilators to emit light, there is usually only one peak of emission, and there is no color mixing [55]. In contrast, the emission of Ln-doped UC nanostructures relies on multiple *f-f* orbital transitions, resulting in an over-reliance on experimental factors for the accuracy and sensitivity of Ln-doped UC nanostructures as biosensors [94,149,150]. Furthermore, there have been many challenges in creating inorganic Ln-doped UC nanostructures-based sensors due to the variations in the aggregation of conjugated dyes and other materials, which create difficulty in controlling the sensor’s readouts [55]. Recently, Huang et al. have shown that TTA UC-based nanostructures are capable of detecting glucose and measuring enzymatic activity related to glucose metabolism, proving that TTA-UC-based nanostructures are viable as background self-standing biosensors. In addition, TTA-UC-based nanostructures also have the potential to act as nanothermometers, monitoring temperature in vivo, which has been found to be useful for medical diagnoses, physiological studies and controllable hypothermia treatments [151]. This property is only possible due to the reliance of TTA UC on the diffusion of component chromophores, which are sensitive to changes in temperature. Recently, Xu et al. have shown that this is possible with the creation of TTA-UC-based nanostructures with temperature-dependent UC luminescence [151]. In conclusion, due to their unique properties, TTA-UC-based nanostructures have several advantages over Ln-doped UC nanostructures, including a high UC quantum yield, low excitation power, temperature sensitivity and controlled readouts. As a result, non-Ln-doped UC nanostructures show enormous potential as bioimaging nanoparticles, biosensors and nanothermometers. Hence, the investigation of new TTA-UC pairs with a high quantum yield, TTA-UC-based MOFs drug carriers and NIR-triggered TTA-UC-based drug delivery systems may be the future focus.

In the TTA-UC process between metal-organic complexes and triplet annihilators, the exchange energy losses will be of the order of hundreds of meVs [152], which limits their quantum yield. Recently, inorganic semiconductor materials, spherical semiconductor quantum dots and low-dimensional materials were found to be efficient triplet sensitizers, and TTA-UC process can still occur when coupled with a specific annihilator [153,154,155,156]. The CdTe nanorod is reported to be a one-dimensional triplet sensitizer. With the addition of a triplet transmitter ligand (9-anthracenecarboxylic acid) and triplet annihilator (9,10-diphenylanthracene), efficient photon upconversion was observed under a low threshold power at 93 mW/cm^2^.

Many UC nanostructures-based core shell MOFs and UC nanostructures-loaded MOFs have been developed as drug carriers and UCL imaging agents, which have a large drug loading efficiency and emit upconversion luminescence. Few MOFs based on TTA-UC were studied. Two MOFs are examples of such systems with a unique structure. Zn-metalated sensitizers are coordinated with Zn_2_ nodes in a paddlewheel fashion to form 2D sheets, and then each sensitizer is connected to five annihilators, which leads to a high TTA-UC efficiency of 1.95% (theoretical maximum = 50%) at an excitation power density of 25 mW cm^–2^ [157]. A new zirconium (Zr)-based UiO-type MOF was constructed from an annihilator linker, [4-((10-(4-carboxyphenyl)anthracene-9-yl)ethynyl)benzoic acid] (H2CPAEBA). This structure allowed for the in situ incorporation of the sensitizer Os(tpyCOOH)_2_^2+^, resulting in efficient triplet sensitization under NIR excitation [158].

Studies on other upconversion nanostructures such as carbon dots have been reported [159,160], but this UCL was questioned because the second-order diffraction light of wavelength λ/2 coexists with the selected light (first-order) of wavelength λ from the monochromators of the spectrofluorometer [161]. This means that if the monochromator is set to 900 nm for excitation, the second-order diffraction light of 450 nm (900 nm/2) would be co-excited on the excitation channel. Under this condition, the emission spectrum that is supposedly excited at 900 nm is actually excited by 450 nm. In addition, the light intensity of this second-order diffraction light (450 nm) caused by the 900 nm setting is much lower than that of the counterpart first-order diffraction light. Based on this, the supposed emission spectrum at 900 nm would have the same peak positions but a lower fluorescence intensity than the spectrum at 450 nm. The comparison of the emission spectrum excited at λ/2 and λ may become a quick method to verify whether or not the UCL of new materials is real without using filters. A similar UCL phenomenon was observed on graphene quantum dots and proved to be artificial for the same instrumental reason [162]. Recently, CoFe_2_O_4_@mSiO_2_ was reported to have UCL [163], but the characteristics of the spectrum between the UCL spectrum and the downconversion spectrum is similar to the former spectra. This may mean that this UCL phenomenon is attributed to the instrument problems and is not the real upconversion. Therefore, the use of light filters and the check of the monochromator might become vital for the study of the upconversion properties of new materials.

## 5. Conclusions

In summary, Ln-doped UC nanostructures have been extensively investigated and can be easily functionalized or modified to load various reagents for therapeutics. The UCL with high SNR is also suitable for imaging-guided diagnosis. TTA-UC-based nanostructures have good excitation and emission tunability, a high quantum yield and a low requirement of laser power, which lower the cost for UCL imaging. The encapsulation of organic sensitizer and annihilator molecule pairs with other drugs into polymeric matrices or micelles is necessary for theranostic applications. Both are suitable for theranostic applications. Most of the theranostic applications are based on the Ln-doped UC nanostructures. These structures can directly form nanocomposite carriers with other polymeric materials, such as PVP, PAA and modified PEG. Multifunctional nanocomposites can also be prepared by incorporating them with other materials, including MOFs, in order to achieve a high drug loading efficiency. Since these materials could convert NIR light to visible and UV light, this high energy emission light could (1) realize UCL imagining, (2) trigger the photosensitizer to generate ROS and (3) trigger the photocleavage reaction of specific functional groups. This review paper has highlighted the application of a UC nanostructure-based theranostic system in tumor-targeted bioimaging and chemotherapy, image-guided diagnosis and phototherapy, NIR-triggered controlled releasing and bioimaging. Furthermore, the challenges of the investigation of TTA UC-based theranostic systems with low laser power and higher biosafety should be addressed in the future.

## Figures and Tables

**Table 1:** Summary of the composition and surface modification strategies of Upconversion nanostructures for biomedical applications and their theranostic applications. UCL: upconversion luminescence, MRI: magnetic resonance imaging, CT: computerized tomography, PTT: photothermal therapy, PDT: photodynamic therapy, PEG: polyethylene glycol, UV: ultraviolet, MOFs: metal-organic frameworks.

**Surface Modification Strategies**	**Composition of Upconversion Nanostructures**	**Therapy**	**Diagnosis**		**Ref.**
			**In Vitro**	**In Vivo Bioimaging**	
Mesoporous silica coating	NaYF_4_:20%Yb,2%Er@NaGdF_4_ NPs	Loading cis-platinum pro-drugs and cytotoxic protein ribonuclease A for RNA-targeted tumor therapy and proteins-based therapies.	HepG2 cells	H22 tumor-bearing Balb/c mice (UCL and T_1_-weighted MRI)	[13]
	NaYF_4_:17%Yb,3%Er NPs	Adsorption of ibuprofen.	HeLa cells	-	[74]
	NaYF_4_:25%Yb,2%Er,0.5%Tm NPs	Rose Bengal and zinc(II)phthalocyanine for PDT.	HeLa cells	-	[75]
	NaGdF_4_:20%Yb,2%Er@NaGdF_4_:30%Nd,10%Yb	Chlorin e6 and merocyanine 540 for PDT.	HeLa cells	U14 tumor-bearing Balb/c mouse (UCL and CT imaging)	[76]
	NaGdF_4_:18%Yb,2%Er@NaGdF_4_:	Rose Bengal for PDT. Conjugation with Linker-Protein G for tumor targeting.	HT-29 cells	-	[77]
Silica and manganese dioxide coating, PEGylated surface	NaGdF_4_:20%Yb,2%Er@ NaGdF_4_:20%Yb NPs	Loading DOX for chemotherapy. Loading chlorin e6 for PDT.	HeLa cells	U14 tumor bearing mice (UCL, T_1_-weighted MR and CT imaging)	[99]
Silica and manganese dioxide coating	NaGdF_4_:19%Yb,1%Er,1%Tm@NaGdF_4_:10%Yb@NaNdF_4_:10%Yb	Chlorin e6 for PDT.	L929 cells and HeLa cells	U14 tumor-bearing Kunming mice (UCL, T_1_-weighted MR and CT imaging)	[100]
Silica and cerium oxide coating, PEGylated surface	NaGdF_4_:20%Yb,1%Tm@NaGdF_4_	Cerium oxide for PDT. DOX for chemotherapy.	L929 cells and HeLa cells	U14 tumor-bearing mice. (UCL, T_1_-weighted MR and CT imaging)	[101]
Silica coating, followed by hyaluronic acid modification	NaYF_4_:20%Yb,2%Er	Titanium dioxide and DOX for sonodynamic therapy and chemotherapy, respectively.	KB and MCF-7 cells	S180 tumor-bearing mice (UCL)	[14]
Silica coating, PEGylated surface	NaYF_4_:24.7%Yb,0.3%Tm NPs	DOX for chemotherapy.Folic acid for tumor targeting.	HeLa cells	HeLa cells of tumor-bearing nude mice (UCL)	[102]
PEGylated	Y_2_O_3_:Yb^3+^/Er^3+^ hollow nanospheres	DOX loaded for chemotherapy.	HeLa cells	UCL imaging in anaesthetized white ICR mice (UCL)	[103]
	NaGdF_4_Yb/Nd@NaGdF_4_:Yb/Er@NaGdF_4_	Rose Bengal for PDT, and Pt(IV) prodrugs for chemotherapy.	A2780 cells	-	[104]
	NaYF_4_:18%Yb,0.6%Tm@NaYF_4_	Resonant excitation ^2^F_5/2_ → ^2^F_7/2_ of Yb^3+^ for PTT.	A375 and HEK 293 cells	A375 Male Balb/c nu/nu mice	[105]
	NaGdF_4_:20%Yb,2%Er@NaGdF_4_:25%Yb,25%Nd	Cerium oxide for PDT. Nanographene oxide for PTT.	L929 cells and HeLa cells	U14 tumor bearing mice (UCL imaging)	[106]
	NaYF_4_:20%Yb,2%Er	protoporphyrin IX for PDT. Conjugate to AS1411 for cancerous cells targeting.	MCF-7 and HeLa cells	-	[107]
	NaYF_4_:27%Yb,2%Er	Phthalocyanine zinc for PDT. Conjugate with Gefitinib (G) to target the ATP binding domain of the tyrosine kinase.	HepG2 cells and HELF cells	-	[108]
PEG and folic acid-modified	NaYF_4_:25%Yb,0.3%Tm NPs	DOX for chemotherapy. MoS_2_ for PDT.	HeLa and HepG2 cells	-	[97]
Stabilization by polyetherimide, followed by PEG modification	NaGdF_4_:40%Yb,0.5%Tm@NaGdF_4_:2%Yb NPs	ZnFe_2_O_4_ for PDT. Pt(IV) prodrugs for Glutathione-mediated cancer cell killing.	HeLa cells	U14 cells (cervical carcinoma cells) of female Balb/c mice (UCL)	[36]
	NaYF_4_:40%Yb,0.5%Tm@NaGdF_4_:2%Yb NPs	Trans-platinum(IV) prodrug triggered by upconverted emission UV light.	HeLa cells	H22 tumor-bearing female Balb/c mice (UCL)	[37]
	NaGdF_4_:17%Yb,3%Er NPs	Platinum(IV) prodrug for chemotherapy. Delivery of siRNA to the silence gene (eukaryotic translation initiation factor 4E).	Hep-2 cells and L929 cells	Anesthetized Balb/C nude mice (UCL)	[38]
Stabilization by polyetherimide, followed by chitosan wrapped surface	NaYF_4_:Yb/Er	Pyropheophorbide a for PDT. Conjugate with RGD peptide c for targeting.	U87-MG cells	-	[39]
Polyetherimide-modified	NaYF_4_:20%Yb,2%Er hollow nanospheres	DOX for chemotherapy.	KB cells	-	[88]
	NaGdF_4_:17%Yb,3%Er NPs	Delivery of bcl-2 siRNA for gene therapy for tumors.	HeLa cells	Anesthetized Kunming mouse (UCL, T_1_-weighted MR and CT imaging)	[91]
Surface coated by TWEEN	NaYF_4_:20%Yb,2%Er @ NaYF_4_ NPs	Doxorubicin (DOX) loaded for chemotherapy.	HeLa cells.	-	[42]
Transferrin-coated	NaYF_4_:30%Gd,18%Yb,2%Er NPs	Protoporphyrin IX for PDT. Magnetically assisted tumor cell targeting.	MDA-MB-231 and HeLa cells	-	[44]
Alpha-cyclodextrin-modified	CaF_2_:20%Yb,2%Er NPs	DOX for chemotherapy.	HeLa cells	Anaesthetized Kunming mouse (UCL and CT imaging)	[109]
Surface functionalized by 15-carboxy-N,N,N-trialkylpentadecan-1-ammonium bromide	NaYF_4_:Yb/Er@ NaGdF_4_ NPs	DOX for chemotherapy. pH responsive.	HeLa cells	-	[110]
Gelatin-modified	BaGdF_5_:20%Yb^3+^,2%Tm^3+^@BaGdF_5_:x%Yb^3+^ Ultra-small NPs	DOX for chemotherapy. pH triggered drug releasing.	HeLa cells	Anesthetized white Kunming mice (UCL)	[111]
Polysaccharide polymer (guar gum)-coated	NaYF_4_:20%Yb,2%Er@ NaYbF_4_ NPs	Rose Bengal for PDT. 5-fluorouracil for chemotherapy. Target releasing in the colon.	HT-29 colon carcinoma cells	-	[43]
Poly(acrylic acid)-modified	NaYF_4_:18%Yb,2%Er@NaYF_4_:10%Yb NPs	DOX for chemotherapy.	MCF-7 cells	H22 tumor-bearing female Kunming mice (UCL)	[34]
Citric acid modification, followed by a growing gold shell on the surface	NaYF_4_:20%Yb,2%Er@NaGdF_4_ NPs	DOX for chemotherapy. Gold shell for PTT.	HeLa cells	-	[33]
Formation of MOFs on the surface	NaYF_4_:20%Yb,1.5%Er,0.5%Tm NPs	DOX and 5-fluorouracil for chemotherapy.	HeLa cells	-	[98]
Formation of MOFs(MIL-53-NH_2_) on the surface, and PEG-functionalized	NaGdF_4_:20%Yb,2%Er@NaGdF_4_:30%Nd NPs	DOX loaded for chemotherapy. Folic acid for tumor targeting.	HeLa cells	-	[90]
Formation of Zr (IV)-based porphyrin MOFs on the core surface	NaYbF_4_:80%Er@NaGdF_4_:20%Yb,2%Er@NaGdF_4_	The composition of MOFs (Zr6 clusters) for PDT. Conjugated with anti-programmed death ligand 1 for immunotherapy.	CT26 cells	CT26 tumor-bearing female BALB/c mice (NIR-II imagining)	[84]
Encapsulation into MOFs (UiO-68-NH_2_)	NaGdF_4_:20%Yb,2%Er@NaGdF_4_:40%Nd,10%Yb NP	Chlorin e6 and Rose Bengal for PDT.	4T1 cells	4T1 tumor-bearing female Balb/C mice	[86]
Encapsulation into nano-phospholipids	NaYF_4_:20%Yb,2%Er NPs.	Loading various PDT reagents.	HeLa cells, KB cells and REF52 cells	-	[112]
Encapsulation into liposomes	NaYF_4_:60%Yb,2%Er	DOX and methylene blue for chemotherapy and PDT, respectively. Conjugated to the anti-HER2 peptide to target breast cancer cells.	SKBR-3 breast cancer cell lines	-	[89]
	NaGdF_4_:20%Yb,2%Er	Loading docetaxel for treating gliomas.	C6 glioma cells	-	[92]
